# Does transversus abdominis function correlate with prone plank and bench bridge holding time in club cricket players?

**DOI:** 10.17159/2078-516X/2022/v34i1a12984

**Published:** 2022-01-01

**Authors:** KD Aginsky, K Keen, N Neophytou

**Affiliations:** Centre for Exercise Science and Sports Medicine, School of Therapeutic Sciences, Faculty of Health Sciences, University of the Witwatersrand, South Africa

**Keywords:** core stability, abdominal muscle thickness, abdominal hollowing, bridging

## Abstract

**Background:**

Bridge and plank holding times are used to evaluate core stability. Transversus abdominis (TA) muscle function is assessed using ultrasound and also provides input on an individual’s core stability.

**Objectives:**

A correlation study comparing TA muscle function with bridge and plank holding time in club cricketers.

**Methods:**

Seventeen male, premier league cricketers (age: 22.1 ± 3.3 years) participated in this study. Ultrasound was used to measure bilateral TA, internal oblique (OI) and external oblique (OE) muscle thickness at rest and during abdominal hollowing. Muscle function was measured by means of a Pearson’s correlation as the change in muscle thickness from rest to abdominal hollowing and compared to holding time of the bench bridge and prone plank (seconds).

**Results:**

TA muscle thickness was preferentially recruited bilaterally (p=0.00001) during abdominal hollowing. No significant correlations were found between TA muscle function and holding time for the bench bridge (dominant (D): r = 0.03 [95% CI:-0.46–0.50]; non-dominant (ND): r = −0.02 [95% CI:−0.50–0.47]) or prone plank (D: r = −0.16 [95% CI:−0.60–0.34]; ND: r = −0.13 [95% CI:−0.57–0.38]).

**Conclusion:**

Prone plank and bench bridge holding times are not correlated with TA muscle function during abdominal hollowing. Core stability cannot rely on a single test to evaluate its effectiveness. In particular, the contribution of the local and global muscle system to ‘core stability’ needs to be evaluated independently. Therefore these tests are not sensitive enough to evaluate the contribution of the local muscle system to the global muscle system in a healthy, pain free, sporting population.

Cricket is a popular sport that requires the player to produce enough power through the kinetic chain to bowl, bat, or field.^[1]^ This energy transfer is aided by strong core muscles that stabilise and support the spine during vigorous movements.^[2]^ The core is made up of abdominal and lumbar muscles that are divided into local and global muscles based on their role in stability and strength.^[3]^ The deeper lying trunk muscles, such as the transversus abdominis (TA) and lumbar multifidus, act as active stabilisers of the lumbar spine, with stabilisation being attained with the bilateral activity of these muscles. ^[1, 2]^

The TA muscle provides segmental stability, postural balance, and contracts to stabilise the body through an increase in intra-abdominal pressure and resisting rotational forces of the spine when forces are applied to the trunk. ^[4]^ With pain, the TA muscle becomes inhibited, which may in turn result in spinal instability, and decreasing one’s ability to control the movement of the trunk during sporting activities. ^[5]^ TA muscle function can be evaluated with the use of ultrasound and is measured as the change in muscle thickness from rest to contraction. The TA is preferentially recruited at 20% to 30% of maximal voluntary contraction (MVC) during abdominal hollowing, ^[6]^ which is achieved by drawing the abdomen towards the spine without tilting the pelvis. ^[7]^ Abdominal hollowing has been found to preferentially recruit the TA when compared to other lateral abdominal muscles.

Spine stability is the interaction between the global trunk muscles (rectus abdominis, external oblique and erector spinae) and the local trunk muscles (TA and deep multifidus), as well as the global stabilisers (quadratus lumborum and internal abdominal oblique). ^[8]^ Stability is achieved by the generation of muscle coactivation patterns and can be measured in many ways. Typically, local muscle function has been assessed using fine wire EMG or ultrasound during simple tasks such as abdominal hollowing. ^[9]^ Global muscle stability is measured during activities, such as the isometric bridge hold test, bench bridge and prone plank activities. In most cases, the test evaluates the individual’s ability to maintain a neutral spine during a static holding position.^[10]^ While there is relatively good reliability for the measurement of local muscle stability ^[4, 11]^, the reliability of the global muscle stability tests are inherently poor. Bridging is a practical and valid method that tests a component of lumbar spine stabilisation. ^[12]^ The bench bridge test is used to assess global core muscle strength, and although the reliability is poor, it is considered to have clinical significance. ^[13]^ Furthermore, it has been postulated by clinicians and trainers that the prone plank and bench bridging trains core stability. However, the relationship between TA muscle function and bridging holding time has not previously been investigated. By assessing this relationship, it could be determined that if one has better TA function they may be able to maintain a bridge exercise for a longer period of time.

Therefore, the aim of this study is to determine whether there is a relationship between the function of the TA muscle and global core function as measured during bridge and plank holding times.

## Methods

### Study design

This was a correlation and descriptive study performed on 17 club cricket players between the ages of 18 to 25 years old. The sample used was one of convenience. Individuals without a history of lower back pain or presently suffering from lower back pain were included in the study. Ethical clearance was obtained from the Human Research Ethics Committee (M130451) at the relevant institution and all cricketers signed informed consent forms prior to testing.

### Body mass, height and fat percentage

Body mass (kg) and stature (m) were measured using a Safeway scale and Seca stadiometer respectively. Seven skinfolds were measured to determine the sum of skinfolds (mm) and body fat percentage (%) using the Durnin and Wormesley formula ^[14]^ [100(4.570/D-4.142) where D = 1.1620-(0.0630x L) and L=log of four skinfold measurements (triceps, biceps, subscapular and suprailiac sites]. A skinfold caliper and measuring tape were used to measure the skinfolds on the right side of the body, with the cricketer standing upright. The seven sites included the triceps, biceps, subscapular, suprailiac, abdomen, mid-thigh and calf.

### Lateral abdominal muscle ultrasound

The lateral abdominal muscles: TA, OI and OE muscles were viewed with a digital ultrasonic diagnostic imaging system (Mindray DP-6600, Shenzhen Mindray Bio-Medical Electronics Co., Ltd., Shenzhen, China) to determine the thickness of the muscles at rest and during abdominal hollowing. Scanning was performed on both the dominant (D) and non-dominant (ND) sides to assess muscle symmetry. Abdominal hollowing was used to assess muscle function of the TA as this task preferentially recruits the TA, with small changes in the OE and OI muscles, respectively. Participants lay supine in the crook-lying position with knees bent to 90°. The transducer head was placed transversely midway between the iliac crest and inferior border of the rib cage in the mid-axillary line, with the medial edge 2.5 cm from the midline, as this allows for all three abdominal muscles to be observed simultaneously. ^[6, 15, 16]^ Once accurate visualisation of all three abdominal muscles at rest was obtained, the image was frozen and the automatic caliper function was used to measure the muscle thickness of the three abdominal muscles (mm). Each measurement was taken as the distance between the edge of the upper and lower inner fascia, represented by the hypoechoic areas on ultrasound, and in three places along the muscle with the average measurement (mm) recorded. The cricketers then performed the abdominal hollowing task at 20–30% of their maximal voluntary contraction without assistance from the spine or pelvis. ^[15]^ They were instructed to breathe in deeply and on exhalation, pull their belly button up and inwards gently towards their spine. Whilst performing this task, the image was again frozen and the measurement of the muscle thickness (mm) taken as before. TA muscle function was assessed by the change in muscle thickness (Δmm) and was calculated as follows: muscle thickness during abdominal hollowing and muscle thickness during rest.

### Prone plank and bench bridge

The bench bridge and prone plank tests were used to assess the cricketer’s core strength. ^[13]^ The bench bridge was performed with the cricketer lying supine on the floor and placing both feet on a bench, with a height of 30 cm. Before performing the prone blank and bench bridge tests, the subjects did a five minute standardised warmup of running drills. Each subject was also familiarised with the tests by performing two planks and two bridges prior to the actual test being recorded. Prior to the beginning of timing, the cricketer lifted his pelvis so that his shoulders, hips, knees and ankles were in a straight line. Once in this position the timer was started. If the cricketer was unable to maintain this body alignment due to discomfort, pain or fatigue, they were given two opportunities to correct their alignment; thereafter the test was terminated, and the time recorded (seconds).

The prone plank position was performed with the forearms flat on the floor and elbows perpendicular to the floor. The body was held parallel to the floor when the participant raised his body up onto the toes and the shoulders, hips and knees were aligned. The back was flat, abdominal muscles were contracted and the buttocks did not lift or drop. The time (seconds) that the participants were able to hold the correct position was recorded. A player was allowed to make two corrections to their body position, after which the test was terminated, and the time was recorded.

### Statistical analysis

Descriptive data were reported as means and standard deviations. Student’s t-test was used to assess the change in thickness from rest to the contraction of TA, OI and OE muscles to investigate whether the TA was preferentially recruited, and to assess muscle thickness symmetry (p<0.05). A Pearson’s correlation was used to assess the relationship between lateral abdominal muscle function and bridge holding time. Statistical significance was taken as 95%.

## Results

Seventeen male club cricketers (batsmen and bowlers) from premier league cricket teams were tested. The mean age was 22.1 ± 3.3 years; mean height was 1.8 ± 0.1 m, and mean weight was 74.8 ± 7.3 kg. The mean BMI was 23.3 ± 1.6 kg/m^2^, placing this group in the normal category of 18.5 to 24.9 kg/m^2^. The sum of skinfolds was 73.5 ± 26.2 mm and the mean body fat percentage was 14.7 ± 4.0%. Dominance was taken as the preferred bowling or batting side. Thirteen players were right dominant and four were left dominant.

### Lateral abdominal muscle thickness

**Fig. 1:**
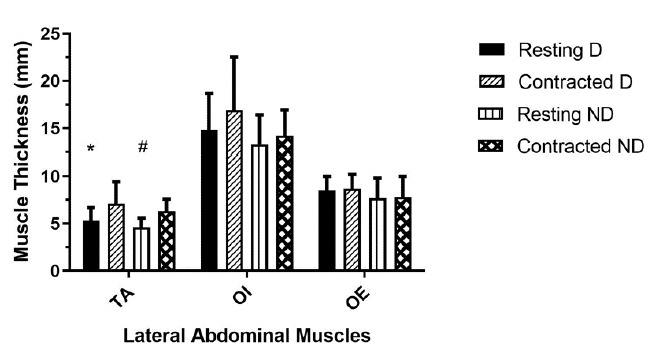
Mean absolute resting and contracted bilateral muscle thickness (mm) of the TA, OI and OE muscles in premier league club cricketers (n =17). TA, transversus abdominis; OI, internal oblique; OE, external oblique; D, dominant; ND, non-dominant. * TA D rest vs. TA D abdominal hollowing (p = 0.00001); # TA ND rest vs. TA ND abdominal hollowing (p = 0.00001).

Dominant and non-dominant muscle thickness at rest and during abdominal hollowing for the TA, OI and OE muscles are shown in Figure 1. When assessing the change in muscle thickness from rest to abdominal hollowing, the TA muscle thickness was found to significantly increase on both the dominant and non-dominant sides (p = 0.00001). Importantly, there was no significant change in the thickness of the dominant and non-dominant OI and OE muscles, indicating that the TA muscle was preferentially recruited during the abdominal hollowing contraction.

### Bench bridge and plank holding times

The mean holding times for the prone plank (n=17) and bench bridge (n=16) are shown in Table 1. One cricketer was unable to successfully complete the bench bridge due to shoulder pain and was thus excluded from this analysis.

### Lateral abdominal muscle thickness vs bridge holding time

There were no significant relationships between the dominant and non-dominant TA muscle function and the amount of time the premier league cricketers were able to hold the bench bridge or prone plank (Table 2). There was also no relationship between change in OI muscle thickness and prone plank or bench bridge holding times.

## Discussion

Change in muscle thickness from rest to contraction (abdominal hollowing) has been shown to be a reliable measure of muscle function for the transversus abdominis.^[6, 9]^ Research has shown that the abdominal hollowing contraction preferentially recruits the TA muscle. ^[15]^ In this study the TA muscle on both the dominant and non-dominant sides showed a significant change in muscle thickness from rest to abdominal hollowing. There was no difference in the change in muscle thickness for the OI and OE muscles bilaterally, thus indicating that the TA muscle was indeed preferentially recruited during the abdominal hollowing task.

There was no relationship between the prone plank and bench bridge holding time and TA muscle function. It has been postulated by clinicians that prone plank and bench bridging are measures of core stability. ^[1, 13]^ The transversus abdominis muscle’s function, as measured by the change in muscle thickness from rest to contraction, is a component of spinal stability when performed at 30% of a maximal voluntary contraction (MVC). ^[11]^ The fact that there were no significant correlations between the prone plank and change in TA muscle thickness or during abdominal hollowing, is likely due to the fact that during the prone plank exercise the global abdominal muscles are primarily recruited ^[17]^, thus making this exercise more for core strength and global stability. Furthermore, the global muscles are recruited at a higher percentage of MVC compared to the local muscle system.

The weak correlations with TA function indicates that it is unlikely that the time the bridging position is held for is associated with deep muscle function, and therefore static bridge tests are not indicative of core stability according to these results. Furthermore, it is more likely that bridging is associated with the global strength of the core, which is supported by the findings of Kong et al. ^[18]^ who found through the use of EMG that the rectus abdominis, external oblique, internal oblique and erector spinae muscles were loaded throughout the prone plank. Stevens et al. ^[19]^ also found that during the unilateral bench bridge the contralateral OE muscle activity was significantly higher than that of the local muscles’ activity, indicating that the bench bridge is more likely to train global core strength than inherent core stability. However, the local and global oblique muscles appear to work simultaneously and may have an important role to play in controlling the neutral spine during bench bridging. ^[19]^ However, it may be that during the prone bridge an athlete needs to perform an abdominal hollowing task in order to increase stability and thus perhaps increase the holding time of the bridge. It is recommended that further research be performed following this method for improved lumbar stabilisation assessment. ^[20]^

As previously mentioned, the local muscle system works at a much lower percentage of MVC, and as all the subjects were pain free, the effect of not having an adequately functioning deep muscle system on core stability is difficult to assess. It is recommended that subjects whose deep muscle system has been affected by lower back pain be assessed to determine this effect.

The sample size of this study was small due to a sample of convenience being tested. Therefore, the ranges, as well as the confidence intervals, show a high amount of variation. Furthermore, the bench bridge and prone plank tests have poor reliability which may affect the accuracy of the measurements and may be confounding factors.

## Conclusion

There was no correlation between the prone plank and bench bridge holding times and TA muscle function. The prone plank and bench bridge tests are not sensitive enough to evaluate the contribution of the local muscle system to the global muscle system in a healthy, pain free population.

## Figures and Tables

**Table 1:** Mean bench bridge and prone plank holding times for club cricketers

	Bench bridge (seconds) (n=16)	Prone plank (seconds) (n=17)
**Mean ± SD**	172.3 ± 109.0	230.4 ± 91.3
**95% CI**	104.1 – 220.6	183.4 – 277.3
**Range**	35.0 – 483.0	103.0 – 422.0

**Table 2:** Correlation between dominant and non-dominant TA muscle function and holding time for the bench bridge and prone plank in premier league club cricketers

Muscle function (Δmm)	Bench bridge (n=16)		Prone plank (n=17)	

	r- value (95% CI)	p-value	r- value (95% CI)	p-value
**Transverse abdominis D**	0.03 (−0.46 – 0.50)	0.92	−0.16 (−0.60 – 0.34)	0.53
**Transverse abdominis ND**	−0.02 (−0.50 – 0.47)	0.95	−0.13 (−0.57 – 0.38)	0.63
**Internal oblique D**	0.26 (−0.18 – 0.70)	0.33	0.24 (−0.29 – 0.64)	0.36
**Internal oblique ND**	−0.36 (−0.47 – 0.50)	0.89	−0.07 (−0.50 – 0.46)	0.79

D, dominant; ND, non-dominant; Δmm, change in muscle thickness.
